# Diagnostic Accuracy of the Modified Alvarado Score and Serum C-reactive Protein in Acute Appendicitis

**DOI:** 10.7759/cureus.73664

**Published:** 2024-11-14

**Authors:** Atta Ul Aleem Khalid, Andrew Quarrell, Anupam Chandran, Tasveer Javed, Nadeem Ahmad

**Affiliations:** 1 General Surgery, Scunthorpe General Hospital, Scunthorpe, GBR; 2 General Surgery, Scunthorpe general hospital, scunthorpe, GBR; 3 General Surgery, Pakistan institute of medical sciences, Islamabad, PAK

**Keywords:** alvarado score, appendicitis, crp, diagnostics, histopathology

## Abstract

Background

The Alvarado score is a diagnostic tool to stratify patients on the likelihood of acute appendicitis based on signs, symptoms, and laboratory values. The validity of this score as compared to other diagnostic measures for acute appendicitis is questionable. The current study addresses the use of a modified Alvarado score (MAS) in conjunction with the widely used acute phase reactant biomarker serum C-reactive protein (CRP) for diagnostic accuracy.

Objective

To determine the diagnostic accuracy in terms of specificity, sensitivity, negative predictive value (NPV), and positive predictive value (PPV) of the combined MAS/CRP keeping histopathological diagnosis of acute appendicitis as a gold standard.

Methods

This is a cross-sectional validation study carried out in the Department of Surgery, Pakistan Institute of Medical Sciences, Islamabad. A total of 230 patients undergoing appendicectomy for appendicitis were included in the study through non-probability consecutive sampling, with positive histology undetermined. Prior to surgery, the preoperative Alvarado score was calculated and CRP was determined. The appendix removed at surgery was subjected to histopathological examination and on the basis of its report patients were postoperatively diagnosed either as positive or negative for acute appendicitis.

Results

The mean age of the patients was 22.66±7.48 years. There were 137 (59.6%) males and 93 (40.4%) females. One hundred eighty-three (79.6%) patients had a positive CRP and 47 (20.4%) had a negative CRP. Alvarado scores were calculated and there were 28 (12.1%) patients with a score of ≤ 6, and 202 with a score of 7-9. The appendix removed at surgery was subjected to histopathological examination. One hundred ninety-five (84.7%) patients were positive for acute appendicitis on histopathology and 35 (15.2%) had normal appendix on histopathology. Among the 195 patients with acute appendicitis 178 (91.3%) had positive CRP/MAS and 17 (87.17%) had negative CRP/MAS. Among the 182 patients with positive CRP/MAS; 178 (97.8%) had acute appendicitis and 4 (2.2%) had normal appendix. Among the 48 patients with negative CRP/MAS; 17 (35.4%) had acute appendicitis and 31 (64.3%) had normal appendix. The calculated sensitivity, specificity, PPV, and NPV were 91.2%, 88.5%. 91.8%, and 64.5%, respectively.

Conclusion

MAS used in combination with CRP is a highly sensitive tool for use in the diagnosis of acute appendicitis and is especially useful in resource-limited healthcare settings and for assistance in decision-making for doctors with less clinical experience.

## Introduction

Appendicitis is one of the most common causes of surgical acute abdomen, referring to sudden onset abdominal pain warranting immediate attention [1]. While the classical presentation of appendicitis is well known, there is significant diagnostic uncertainty as the classic combination of signs and symptoms rarely occurs so clearly [2]. The diagnostic uncertainty in this condition is highlighted by the relatively high negative appendicectomy rate in certain populations including women of childbearing age [3].

**Figure 1:**
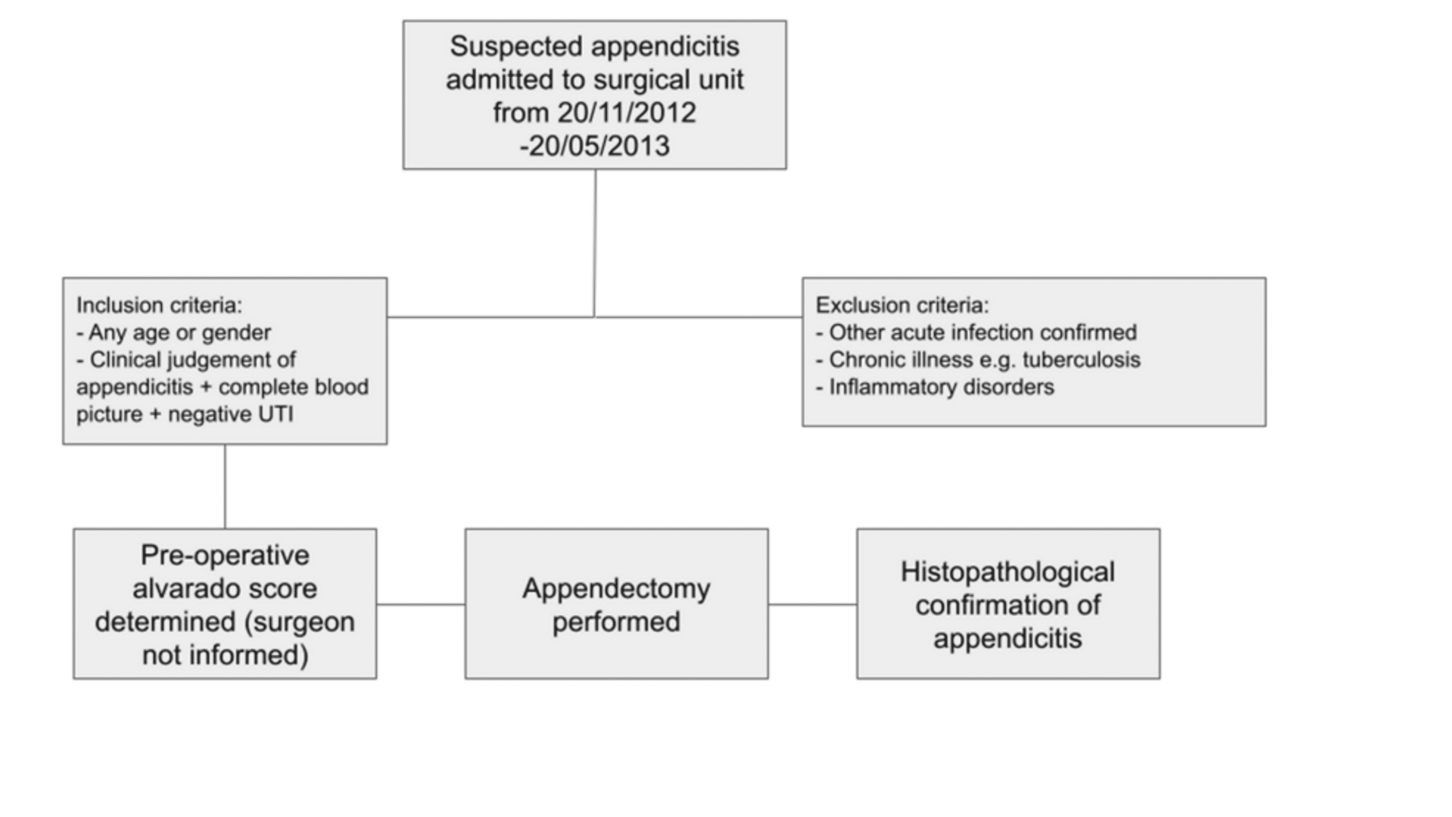
Flowchart of patient journey through the study.

Attempts to increase the diagnostic accuracy of acute appendicitis include predictive scoring systems, laboratory-aided diagnostics, computed tomography scanning, ultrasonography, and diagnostic laparotomy [4,5]. Diagnostic scoring systems are used as a method of quantifying clinical judgment metrics in an attempt to improve diagnostic accuracy. The most prominent of these is called the Alvarado score [6] (Figure 1) which combines the signs, symptoms, and leukocyte changes seen in acute appendicitis. Used in isolation, the score has shown to be useful for junior colleagues but does not necessarily outcompete clinical judgment, especially with experienced clinicians [7]. There may also be some role for its use in avoiding unnecessary radiation from CT scanning, but the evidence on this is inconclusive and may only be purposeful at particularly high Alvarado scores [8].

C-reactive protein (CRP) is an acute phase reactant strongly associated with infection; significant rises can be strongly associated with significant intraabdominal bacterial infections such as those causing appendicitis [9]. A rise in CRP however is non-specific and is more useful either used in combination with other diagnostic measures such as clinical presentation or radiological imaging. CRP levels may also provide benefits by ruling out people who do not have appendicitis, particularly when laboratory values are low 12 hours after the onset of symptoms [10].

The aim of this study is to determine the preoperative clinical accuracy of combining the modified Alvarado score (MAS) with CRP measurements. Our hypothesis is that combining MAS with serum CRP measurements will be better at predicting appendicitis than MAS alone. Previous studies have indicated that the use of MAS alone is insufficient as a diagnostic tool in clinical practice [11,12]. This cross-sectional validation study highlights the benefits of combining two relatively low-intensive diagnostic variables assessing outcomes in terms of sensitivity, specificity, and NPV and PPV values. Histopathological confirmation is used as a gold standard diagnosis with which these values were determined. The current study reinforces the benefits of using CRP and MAS in combination when predicting acute appendicitis. Our data contributes to the body of literature by showing a greater specificity when combining CRP and MAS than previous studies have shown.

## Materials and methods

Definitions

Acute appendicitis was confirmed on histopathology only if there was involvement in the muscularis layer of the appendix. In all cases, at least two transverse sections from the proximal half and a lone longitudinal section from the distal half were studied. Histopathological diagnosis was based on the infiltration of leukocytes in the mucosa and lamina propria.

Alvarado score was used in its modified form (MAS) which omits neutrophil shift to the left, scoring patients out of 9 (for parameters). MAS patients were grouped as follows:

Score ≤6 = no for appendicitis

Score 7-9 = yes for appendicitis

CRP: considered positive if the value is > 6 mg/L.

Study design

The current study is a cross-sectional validation research project performed in the Department of Surgery, Pakistan Institute of Medical Science, Islamabad. The study was conducted from 20th November 2012 to 20th May 2013.

A sample size of 230 patients with suspected acute appendicitis was studied. Two hundred thirty patients were deemed a suitable sample size maintaining a 95% confidence interval and a desired precision of 10%, equivalent to similar scale studies [12].

Sample selection

Inclusion criteria were patients of any age/gender undergoing appendicectomy on the basis of surgical clinical judgment, a negative urinary tract infection (UTI) result on dipstick testing, and a complete bloodwork picture.

Patients were excluded who concomitantly presented with a known cause for raised CRP/inflammatory markers including acute infections (e.g., sore throat, UTI), chronic infections like tuberculosis, and inflammatory disorders such as rheumatoid arthritis and systemic lupus erythematosus. Figure 1 highlights the patient journey from selection in the study through to operation and histopathological determination of appendicitis or not.

Data collection procedure

Informed written consent was taken from patients included in the study. Inclusion and exclusion criteria applied to patients as outlined above. The study was prospective and taken from patients admitted to the surgical unit for acute appendicitis who later underwent appendicectomy, with Alvarado score and CRP determined preoperatively. The decision to operate was not affected by the MAS or CRP levels and the estimated score was not revealed to the operating surgeon. A potential source of error is the similarity between a surgeon’s clinical judgment and the Alvarado score. Consecutive sampling was used to reduce errors in the selection procedure. Patients were excluded with longstanding inflammatory conditions and other acutely diagnosed causes of raised CRP to avoid these confounding variables.

Data analysis

Results were analyzed using SPSS (V.12). Mean and standard deviation were used to present numerical values, e.g., age and Alvarado score. Frequency and percentages were presented for categorical variables. The validity of positive CRP and positive MAS was calculated using a 2×2 table. Descriptive statistical outputs of specificity, sensitivity, positive, and negative predictive values (PPVs and NPVs) were derived from the relative proportions of individuals with pre-test predictors (MAS and CRP) compared to gold standard outputs (histopathological determination of positive or negative acute appendicitis).

## Results

Demographics

**Figure 2:**
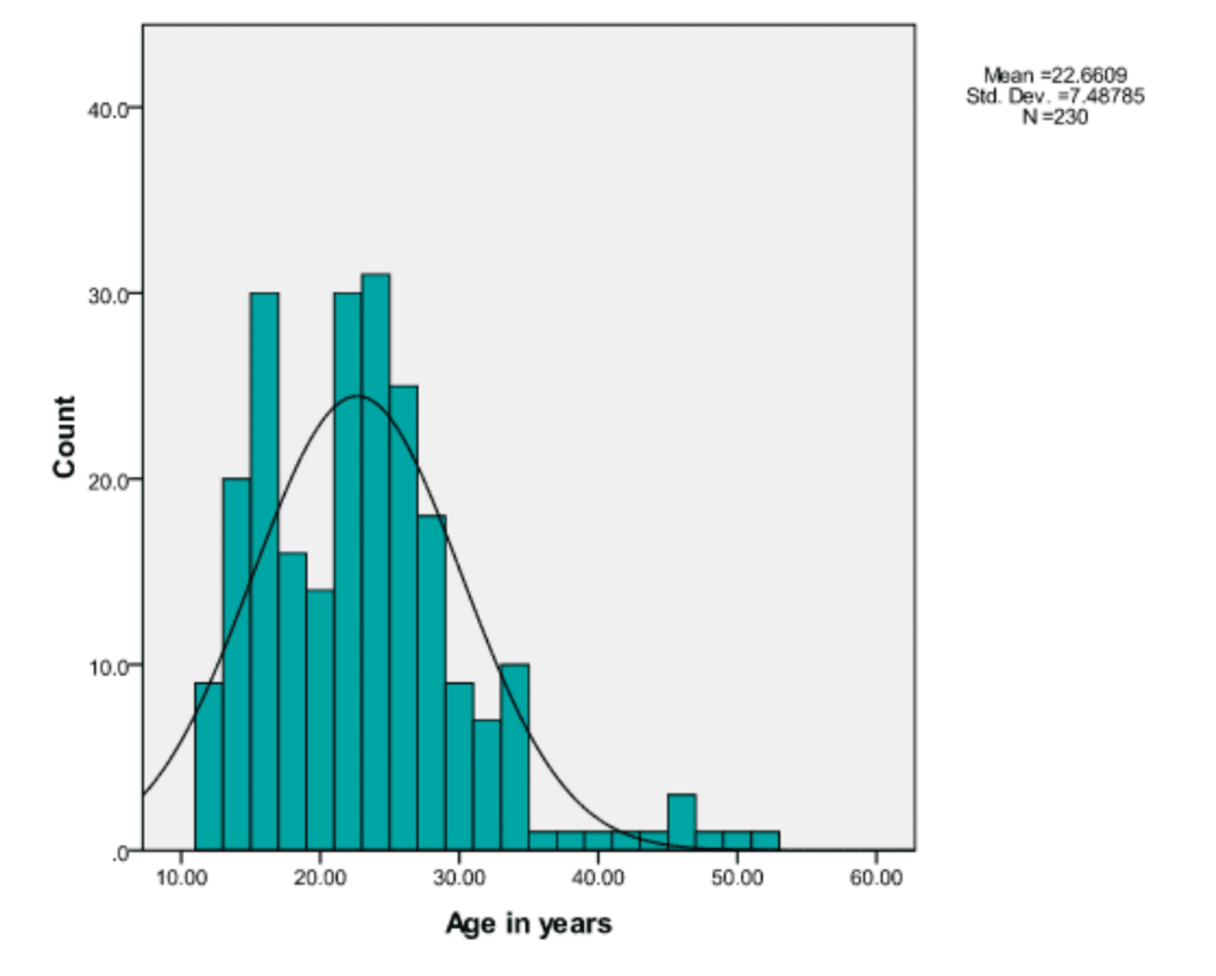
Age distribution

The age of the patients ranged from 12 to 52 years with a mean age of 22.66±7.48 years. The median and mode ages were 22 and 15 years, respectively. There were 137 males (29.6%) and 93 females (40.4%). The difference in age (years) with regard to sex was statistically nonsignificant (p=0.908). The age distribution can be seen in Figure 2.

CRP

CRP was measured using a rapid latex agglutination slide test for the in vitro determination of CRP in the sample. The detection limit is approximately 6 mg/L, and marked agglutination indicated a CRP of more than 6 mg/L. A smooth, homogenous, milky suspension indicated a CRP of less than 6 mg/L.

The CRP was measured using qualitative analysis. One hundred eighty-three (79.6%) of patients had a positive CRP and 47 (20.4%) had a negative CRP.

Alvarado score

**Figure 3:**
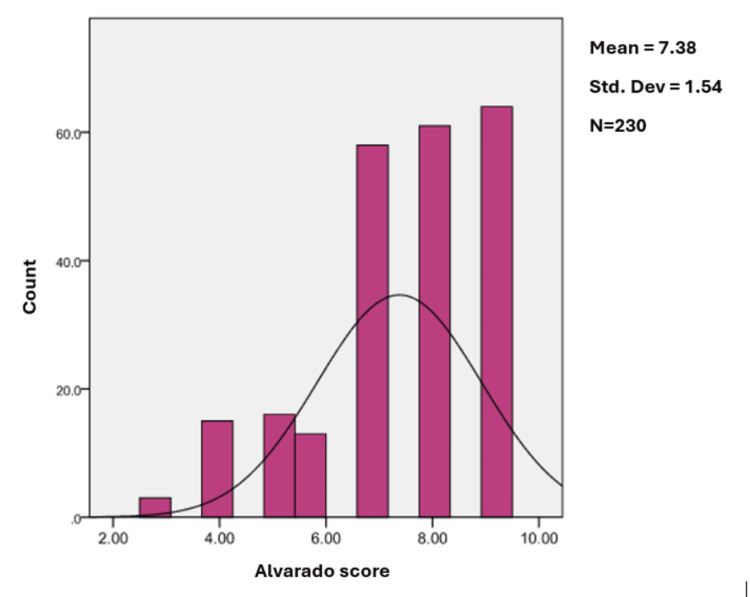
Distribution of Modified Alvarado Score (MAS)

The preoperative MAS of all the patients included in the study was determined and calculated as per Table 1. Twenty-eight patients (12.1%) had a score of ≤6 and thus were assigned unlikely appendicitis by Alvarado score. Two hundred two patients (87.8%) had a score of 7-9 and were thus assigned likely appendicitis. Alvarado scores were 3, 4, 5, 6, 7, 8, and 9 in 3 (1.3%), 15 (6.5%), 16 (7%), 13 (5.7%), 58 (25.2%), 61 (26.5%), and 64 (27.4%) patients, respectively. Alvarado score distribution can be seen in Figure 3.

**Table 1:** The Modified Alvarado Score

Category	Variable	Score
Symptoms	Migratory right iliac fossa pain	1
	Anorexia	1
	Nausea/Vomiting	1
Signs	Right iliac fossa pain (RIF)	2
	Rebound tenderness in RIF	1
	Elevated temperature (>37.5)	1
Investigation	Leukocytosis (>10,000 WBC per microliter)	2
Total Score		9

Histopathology

Following criteria for confirmation as followed by protocols outlined in “materials and methods”, 195 (84.7%) patients were positive for acute appendicitis on histopathology, and 35 (15.2%) had a normal appendix on histopathology.

CRP and MAS

Among the 195 patients with acute appendicitis 178 (91.3%) had positive CRP/MAS and 17 (87.17%) had negative CRP/MAS. Among the 35 patients without acute appendicitis, 4 (11.4%) had positive CRP/MAS and 31 (88.57%) had negative CRP/MAS. This difference was statistically significant, i.e., p =0.00.

Conversely among the 182 patients with positive CRP/MAS; 178 (97.8%) had acute appendicitis and 4 (2.2%) had normal appendix. Among the 48 patients with negative CRP/MAS; 17 (35.4%) had acute appendicitis and 31 (64.3%) had normal appendix.

Diagnostic accuracy of CRP and MAS for acute appendicitis

The summary statistics for the combined CRP/MAS in terms of sensitivity, specificity, PPV, and NPV are given in Table 2.

**Table 2:** 2×2 table summary statistics for the combined MAS/CRP predictive outcomes. MAS: modified Alvarado score; PPV: positive predictive value; NPV: negative predictive value; CRP: C-reactive protein; TP: true positive; FP: false positive; FN: false negative; TN: true negative

		Patients with acute appendicitis confirmed on histopathology		
		Positive histopathology	Negative histopathology	
CRP/MAS	Positive CRP/MAS	178 (TP)	4 (FP)	PPV = 97.8%
	Negative CRP/MAS	17 (FN)	31 (TN)	NPV = 64.5%
		Sensitivity = 91.2%	Specificity = 88.5%	Accuracy (TP+TN) = 90.86%

Sensitivity

The probability that an individual who has positive CRP/MAS indeed has acute appendicitis. It was calculated to be 91.2%.

Specificity

The probability that an individual who does not have positive CRP/MAS did not have acute appendicitis. It was calculated to be 88.5%.

Positive Predictive Value (PPV)

The predictive value positive (PV+) of a screening test is the probability that a person has the disease (acute appendicitis) given that the test is positive (positive CRP/MAS). It was calculated to be 91.8%.

Negative Predictive Value (NPV)

The predictive value negative (PV−) of a screening test is the probability that a person does not have the disease (normal appendix) given that the test is negative (negative CRP/MAS). It was calculated to be 64.5%.

Diagnostic accuracy of MAS and CRP alone for acute appendicitis

The calculated sensitivity, specificity, PPV, NPV, and overall diagnostic accuracy of MAS alone were 98.5%, 71.4%, 95%, 89.2%, and 94.35%, respectively. The calculated sensitivity, specificity, PPV, NPV, and overall diagnostic accuracy of CRP alone were 91.8%, 88.5%, 97.8%, 65.9%, and 91.3%, respectively.

Please see Table 3 for a summary of the statistics grouped by MAS/CRP combination, MAS alone, and CRP alone categories.

**Table 3:** Descriptive statistics of the predictive capabilities of MAS/CRP score, MAS score alone, and CRP used alone. MAS: modified Alvarado score; PPV: positive predictive value; NPV: negative predictive value; CRP: C-reactive protein

	Sensitivity	Specificity	PPV	NPV	Overall accuracy
MAS/CRP	91.2%	88.5%	91.8%	64.5%	90.86%
MAS alone	98.5%	71.4.0%	95.0%	89.2%	94.35%
CRP alone	91.8%	88.5%	97.8%	65.9%	91.3%

## Discussion

We conducted a cross-sectional validation study carried out at the Department of Surgery, Pakistan Institute of Medical Sciences, Islamabad to determine the diagnostic accuracy of CRP plus MAS (7-9) in the diagnosis of acute appendicitis keeping histopathology as the gold standard. A total of 230 patients undergoing appendicectomy for suspected acute appendicitis were included in the study. The mean age of the patients was 22.66±7.48 years. There were 137 (29.6%) males and 93 (40.4%) females. One hundred eighty-three (79.6%) patients had a positive CRP and 47 (20.4%) had a negative CRP. Alvarado scores were calculated and there were 28 (12.1%) patients with a score of ≤ 6, and 202 with a score of 7-9. One hundred ninety-five (84.7%) patients were positive for acute appendicitis on histopathology and 35 (15.2%) had normal appendix on histopathology. Among the 195 patients with acute appendicitis 178 (91.3%) had positive CRP/MAS and 17 (87.17%) had negative CRP/MAS. Among the 182 patients with positive CRP/MAS; 178 (97.8%) had acute appendicitis and 4 (2.2%) had normal appendix. Among the 48 patients with negative CRP/MAS; 17 (35.4%) had acute appendicitis and 31 (64.3%) had normal appendix.

Diagnosis of acute appendicitis is generally established by the surgeon’s clinical impression, and for experienced clinicians, this often outcompetes the Alvarado score [7]. Despite this, the negative appendicitis rate may still be as high as 10-25% [13]. This highlights the importance of accurate diagnosis, especially for patients who may suffer adversely from the outcomes/complications of surgery.

In 1986, Alvarado constructed a 10-point scoring system which is also remembered by the acronym MANTRELS for the diagnosis of acute appendicitis, which recommends laparotomy for all patients with a score of 7 or more [14]. The modification of the score as used in this study pertains to how these results may be more applicable in lower-income settings where a differential for the leukocytosis may not be possible to obtain. While the study here uses the Alvarado score, it should be noted that other scores such as the adult appendicitis score may outcompete Alvarado especially in reducing the need for further CT scanning and identifying safe patients for discharge [15]. Further studies may benefit from using a combination of CRP and these other scores, directly compared to the MAS/CRP diagnostic.

Laboratory studies are a key diagnostic measure in the diagnosis of acute appendicitis, but no single test is definitive. The CRP concentration in the blood reflects ongoing tissue inflammation and is an accurate biomarker of the acute phase inflammatory response [16].

The current study has shown that CRP used in combination with MAS can yield a sensitivity of 91.2% and a specificity of 88.5%. This shows a similar increase in sensitivity compared with Sheikh et al. [17] when combining MAS and CRP in a Nigerian population. Sheikh et al. showed an increase in sensitivity and specificity when combining MAS with CRP in a population that also utilized the modified version of the Alvarado score that does not require a leucocyte differential hence more applicable to low-resource healthcare settings. Comparatively, our study shows the MAS/CRP predicting specificity better than Sheikh et al. (88.5% in our study versus 54%).

It is important to consider that the combination of MAS/CRP may not outcompete the performance of good clinical judgment for experienced general surgeons. Pruekprasert et al. performed a study on a similar population size (231 patients) and compared the surgeon’s clinical diagnosis with the Alvarado score and showed the score had a lower sensitivity (79%) and specificity (62%) when compared to the surgeon clinical judgment [18]. Nevertheless, our study shows that the combination of MAS/CRP yields significantly better sensitivity and specificity as compared to clinical judgment and provides further evidence for the argument of using diagnostic factors in combination when deciding on a diagnosis of acute appendicitis. In this study, CRP with the best clinical judgment in experienced clinicians does outcompete the MAS/CRP combination. Thus, a limitation of this work is that the MAS loses its potency as a predictive tool the more experience a surgeon gains.

We conclude that MAS in combination with CRP levels is a helpful diagnostic tool for the decision to operate in acute appendicitis, especially for junior colleagues with less experience in making clinical judgments. Our study is limited by the relatively young age of the cohort (maximum 52 years old) and the restriction to a Pakistani population. The study does, however, use simple metrics with basic laboratory investigations that do not require a white blood cell differential or scanning equipment, thus making the research applicable to healthcare settings globally without these facilities. In terms of changing practice, our study does not provide evidence that senior, experienced surgeons should use the Alvarado score in place of their own clinical judgment. We do however propose that the use of the score can benefit from being combined with a positive CRP result, especially in terms of facilitating a more specific metric compared to using the score alone. We can conclude that the score itself is a useful tool when the wider context is appreciated.

## Conclusions

Our study showed that in patients with high MAS and raised CRP levels, the probability of acute appendicitis was high. In conjunction, adding CRP to the MAS improves the specificity for correctly identifying individuals without appendicitis. We believe that the use of the MAS in compunction with serum CRP measurements can be of benefit for patients presenting with suspected appendicitis especially where objective clinical judgments are required. These investigations are not costly to the patient, are non-invasive, and do not require any sophisticated equipment or technical expertise. Moreover, the results of these parameters can be obtained rapidly thus reducing unwanted explorations and preventing complications (perforation, abscess) in a timely manner.
